# The production and characterization of a new active lipase from *Acremonium alcalophilum* using a plant bioreactor

**DOI:** 10.1186/1754-6834-6-111

**Published:** 2013-08-01

**Authors:** Eridan Orlando Pereira, Adrian Tsang, Tim A McAllister, Rima Menassa

**Affiliations:** 1 Agriculture and Agri-Food Canada, 1391 Sandford Street, London, ON N5V 4T3, Canada; 2 Department of Biology, The University of Western Ontario, London, ON N6A 5B7, Canada; 3 Centre for Structural and Functional Genomics, Concordia University, Montreal, Quebec H4B 1R6, Canada; 4 Agriculture and Agri-Food Canada, Lethbridge Research Centre, Lethbridge, AB T1J 4B1, Canada

**Keywords:** Lipase, Acetylxylan esterase *Acremonium alcalophilum*, *Nicotiana benthamiana*, Heterologous expression

## Abstract

**Background:**

Microorganisms are the most proficient decomposers in nature, using secreted enzymes in the hydrolysis of lignocellulose. As such, they present the most abundant source for discovery of new enzymes. *Acremonium alcalophilum* is the only known cellulolytic fungus that thrives in alkaline conditions and can be cultured readily in the laboratory. Its optimal conditions for growth are 30°C and pH 9.0-9.2. The genome sequence of *Acremonium alcalophilum* has revealed a large number of genes encoding biomass-degrading enzymes. Among these enzymes, lipases are interesting because of several industrial applications including biofuels, detergent, food processing and textile industries.

**Results:**

We identified a *lipA* gene in the genome sequence of *Acremonium alcalophilum*, encoding a protein with a predicted lipase domain with weak sequence identity to characterized enzymes. Unusually, the predicted lipase displays ≈ 30% amino acid sequence identity to both feruloyl esterase and lipase of *Aspergillus niger*. LipA, when transiently produced in *Nicotiana benthamiana*, accumulated to over 9% of total soluble protein. Plant-produced recombinant LipA is active towards *p*-nitrophenol esters of various carbon chain lengths with peak activity on medium-chain fatty acid (C8). The enzyme is also highly active on xylose tetra-acetate and oat spelt xylan. These results suggests that LipA is a novel lipolytic enzyme that possesses both lipase and acetylxylan esterase activity. We determined that LipA is a glycoprotein with pH and temperature optima at 8.0 and 40°C, respectively.

**Conclusion:**

Besides being the first heterologous expression and characterization of a gene coding for a lipase from *A. alcalophilum*, this report shows that LipA is very versatile exhibiting both acetylxylan esterase and lipase activities potentially useful for diverse industry sectors, and that tobacco is a suitable bioreactor for producing fungal proteins.

## Background

*Acremonium alcalophilum* is a rare cellulolytic fungus that thrives in alkaline environments and can be readily cultured in the laboratory. Isolated from composted pig manure, it grows optimally at 30°C and a pH 9.0-9.2 [1]. Recently, the genome of *A. alcalophilum* was sequenced (http://genome.jgi-psf.org/Acral2/Acral2.home.html), providing a rich genetic resource for the identification of enzymes that potentially function under alkaline conditions.

Lipases (EC 3.1.1.3) are one of the most versatile enzyme classes. They are known to have a number of unique characteristics such as substrate specificity, region-specificity, and chiral selectivity [2]. These enzymes belong to a group defined as carboxylesterases that catalyze the hydrolysis of long and short chain acyl esters [3,4]. Due to their versatility, the use of lipases as biocatalysts has enormous potential to reduce energy requirements and environmental problems in diverse industries. One example is in the detergent industry where fat stains are difficult to remove at low temperatures. By adding lipases that are active at lower temperatures in detergent formulations, energy consumption is reduced as well as the wear and tear of textile fibers. As biocatalysts, lipases could also replace synthetic detergents, which can cause environmental problems such as eutrophication [5]. These advantages have been explored by several other industries including the textile, food, pulp and paper, fat and oleochemical, pharmaceutical and more recently biofuel industries [6]. With the myriad of potential applications, new expression technologies are needed to meet the anticipated demand and lower the cost of lipase production.

Although lipases are known to occur in a diversity of organisms, including animals, plants and microbes, the current industrial production of these enzymes relies almost exclusively on microbial-based expression systems. Filamentous fungi such as *Aspergillus niger* and *Trichoderma reesei* are the workhorses of industry for the production of extracellular enzymes. However, fungi can hyperglycosylate recombinant proteins resulting in reduced activity [7]. An alternative approach is to use plants as bioreactors, transferring the corresponding genes from microorganisms into plants and producing them at economically acceptable levels [8]. Plants offer several advantages over microbes as an alternative expression system for enzymes. These bioreactors utilize sunlight as source of energy and sequester CO_2_ during photosynthesis. Furthermore, they can undertake post-translational modification of enzymes such as glycosylation or formation of disulfide bridges, alterations that are often essential for enzyme stability and sustained activity.

To expand our knowledge of lipases with industrial potential and to develop a new production system, we used heterologous expression in tobacco to produce and characterize a lipase from *Acremonium alcalophilum*. We analyzed the enzyme’s yields, post-translational modification and determined its biochemical characteristics.

## Results

### Identification of the *Acremonium alcalophilum* lipase

**Figure 1:**
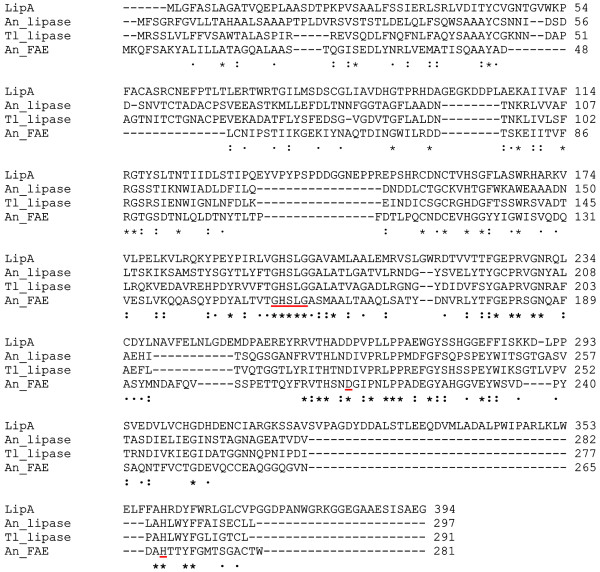
**Alignment of amino acid sequences of LipA from *Acremonium alcalophilum *with other lipases and feruloyl esterase from various microorganisms.** The amino acid sequence of LipA was compared with the lipase amino acid sequences of *Aspergillus niger* (An_lipase), *Thermomyces lanuginosus* (Tl_lipase); and ferulic acid esterase amino acid sequence of *Aspergillus niger* (An_FAE). Identical residues among the four enzymes are marked with *asterisks*. Residues with conserved and semiconserved substitutions are indicated with *colons* and *dots*, respectively; the conserved GXSXG motif and putative residues of the catalytic triad are underlined in red.

With the recent genome sequencing of the alkaliphilic fungus *Acremonium alcalophilum*, over 200 genes encoding biomass-degrading enzymes were identified. Analysis *in silico* of the genome revealed a hypothetical protein (protein ID 1062717 of the *A. alcalophilum* v2.0 database) exhibiting a lipase_3 domain (IPR002921) and weak sequence identity with biochemically characterized fungal proteins. For example, pairwise amino acid sequence alignment using ClustalW2 (http://www.ebi.ac.uk) revealed 25% identity to the triglyceride lipase of *Aspergillus niger* (Accession no. DQ680030, [9]), 24% identity to the lipase of *Thermomyces lanuginosus* (Accession no. O59952, [10]), and 23% identity to the characterized *A. niger* feruloyl esterase (Accession no. O42807.1, [11]) (Figure 1). Therefore, this novel enzyme was provisionally designated LipA*.* However, LipA displays some sequence similarity to uncharacterized enzymes such as 57 and 61% identity to the putative lipases of *Glomerella graminicola* M1.001 (Accession no. EFQ29359.1), and *Verticillium dahliae* VdLs.17 (Accession no. EGY21687.1) respectively, and 40% identity to the putative feruloyl esterases of *Trichophyton equinum* CBS 127.97 (Accession no.EGE08976.1) and *Arthroderma otae* CBS 113480 (Accession no. XP 002843470.1). Taking into consideration the low sequence similarity to two different enzyme activities, and that sequence-based analysis only provides a presumptive compositional and functional blueprint of the gene analyzed, which in some cases may lead to erroneous assignment of substrate specificity [2], we assessed LipA for both lipase and carbohydrate esterase activity.

### Molecular analysis of plant-made LipA

The open-reading frame of the gene encoding LipA is 1128 bp long and encodes a protein of 376 amino acids, with a calculated molecular mass of 41.4 kDa. The deduced amino acid sequence contains one putative *N*-glycosylation site, suggesting that glycosylation could be important for enzyme functionality.

**Figure 2:**
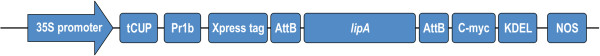
**Schematic representation of the *lipA *expression cassette.** 35S promoter, 35S promoter from Cauliflower Mosaic Virus 35S gene; NOS, nopaline synthase terminator; tCUP, tobacco crypric upstream promoter translation enhancer; Pr1b, tobacco pathogenesis related 1b protein secretory signal peptide; Xpress^TM^, epitope tag (Invitrogen); attB1 and attB2, Gateway® recombination sites; C-myc, detection/purification tag; KDEL, endoplasmic reticulum retrieval tetrapeptide. Schematic not drawn to scale.

**Figure 3:**
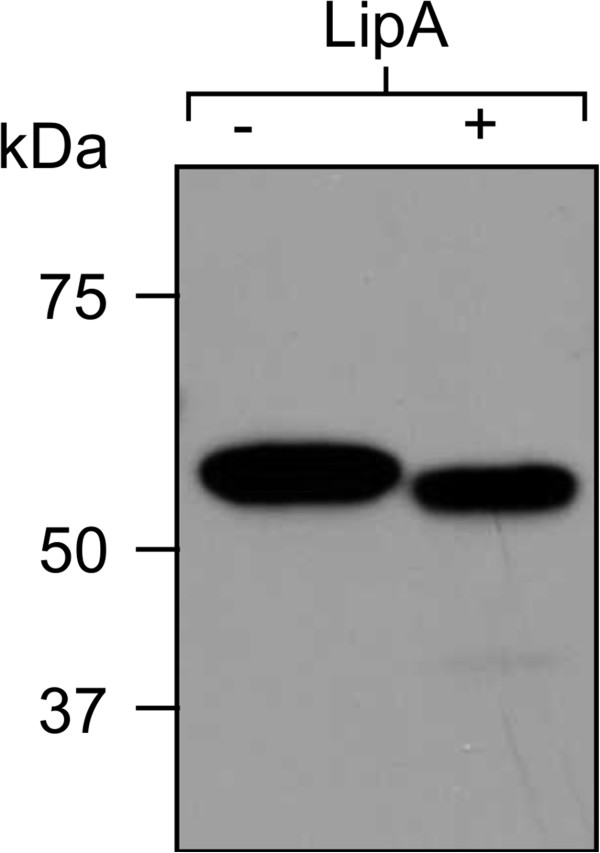
**Transient expression and purification of LipA in *Nicotiana benthamiana *leaves. (A)** Gel code blue stained SDS-PAGE analysis of protein purification from *N. benthamiana* leaves co-infiltrated with LipA and the silencing suppressor p19. Leaf discs were collected 4 days post infiltration (dpi). Lane 1: Extract flowthrough, Lane 2 to 4: Washes and Lane 5: eluted LipA. **(B)** Western-blot analysis of a gel loaded with 5 μg total soluble protein. Lane 1: LipA from plant extract, lane 2: wt plant extract, lane 3 to 5: C-myc standard (24, 72, and 216 ng respectively).

**Figure 4:**
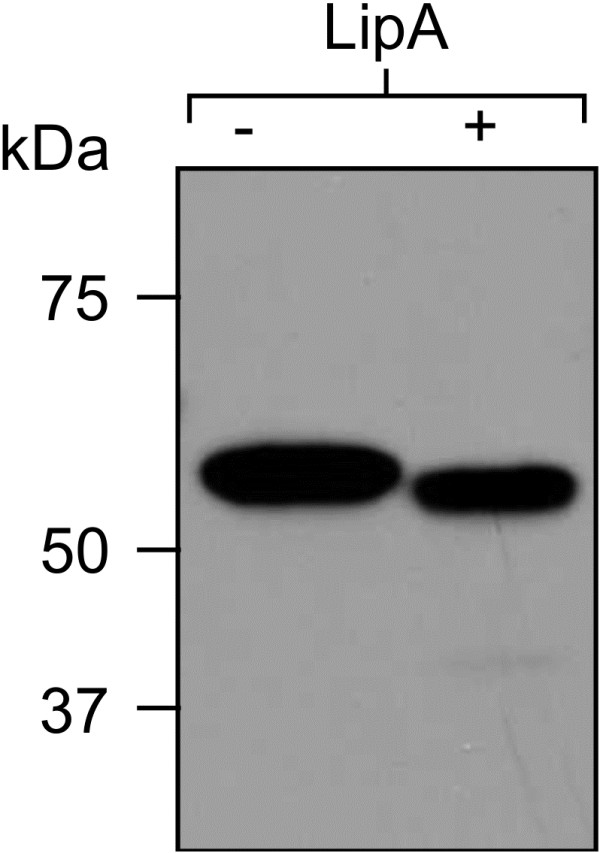
**Deglycosylation analysis of plant-expressed LipA enzyme.** Purified recombinant LipA protein was incubated overnight in the absence (−) or presence (+) of PNGase F, analyzed by SDS-PAGE under reducing conditions, and subjected to western-blot analysis with anti-C-myc antibody.

To characterize LipA properties, we attempted to over-express the *lipA* gene in *A. niger*, and in the plant *Nicotiana benthamiana.* We were unsuccessful in obtaining LipA expression in *A. niger* (data not shown), but obtained high expression in *N. benthamiana* in transient co-expression of the *lipA* gene (Figure 2) with the suppressor of posttranscriptional gene silencing p19 [12]. Crude extracts as well as C-myc purified proteins from pooled samples from five different plants were resolved by SDS-PAGE and GelCode™ Blue staining and were confirmed by western blot analysis with monoclonal antibodies against the fused C-myc tag. This analysis showed that the plant-produced LipA protein has a molecular mass of about 56 kDa (Figure 3a and b). The observed band was larger than the predicted theoretical molecular weight of 46 kDa as the tags add about 5 kDa to the 41 kDa protein. As LipA has a putative *N*-glycosylation site, a deglycosylation experiment was performed by digesting LipA with *N*-glycosidase F (PNGase F). This led to a small downshift in the band size (Figure 4). However, even with deglycosylation, the molecular mass (46 kDa) predicted from the cDNA was substantially lower than that determined by SDS-PAGE. Since intrinsic charge can lead to anomalous migration on SDS-PAGE [13], this difference may reflect the acidic nature of LipA protein, which has an estimated pI of 4.9.

Based on densitometry analysis of western blot results, LipA accumulated to 9.3% of total soluble protein (TSP) (Figure 3B) which corresponds to approximately 2 g of enzyme per Kg of fresh leaf weight. LipA accumulation in our experiments was higher than most *in planta*-expressed glycosyl hydrolases described in the literature [14]. Although a few exceptions with higher expression levels have been described [14], to our knowledge, this is the first time an enzyme with the characteristics presented here has been produced in plants at such high levels.

### Effects of pH and temperature on recombinant LipA activity

**Figure 5:**
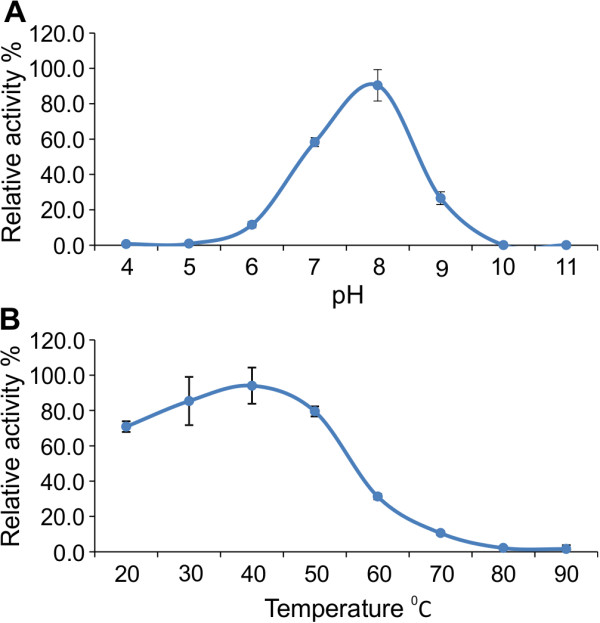
**Effect of pH and temperature on LipA enzyme activity. (A)** The influence of pH on activity was determined using 1 mM *p*NP-caprylate in 100 mM buffers (pH 4.0-5.0 acetate buffer, pH 6.0-9.0 phosphate buffer, pH 10.0-11.0 carbonate buffer) at 40°C for 30 min. **(B)** The influence of temperature was determined in a range from 20-90°C using 1 mM *p*NP caprylate in 100 mM phosphate buffer pH 7.0 for 30 min. The test was performed in triplicate and the average values were transformed in the relative activity (%).

As the goal of this study was to identify and characterize alkaliphilic enzymes for potential application in industrial processes, temperature and pH optima are important characteristics for the efficient usage of this enzyme. Purified plant-produced LipA was used to study the effect of pH and temperature using *p*-nitrophenyl caprylate as substrate. The optimal pH of purified LipA under the standard assay conditions was found to be pH 8.0 (Figure 5a). The enzyme showed maximum lipase activity at 40°C with broad thermostability ranging from 20 C to 50°C (Figure 5b).

### Substrate specificity of LipA esterase

**Figure 6:**
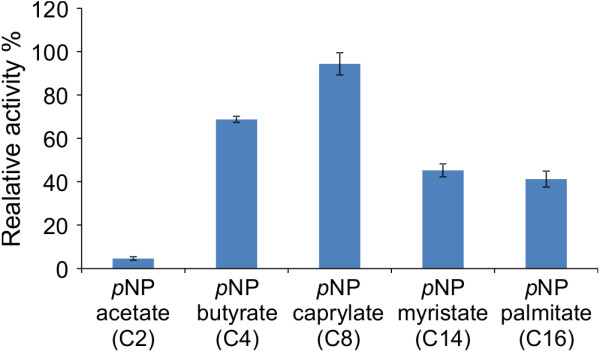
**Substrate specificity of the lipase from *Acremonium alcalophilum *towards *p*-Nitrophenol fatty acid esters with varied acyl chain lengths. Values are means ± SD (n = 3).**

As the gene of interest possessed a sequence similar to that of other genes coding for lipases and feruloyl esterases we evaluated the specificity/preference of LipA for a variety of fatty acid and hydroxycinnamate ester substrates. To investigate the substrate specificity of LipA, the hydrolyzing activity of the purified enzyme was determined at its pH and temperature optima of pH 8.0 and 40°C, respectively. The substrates analyzed included *p*NP esters of various carbon chain lengths, methyl cinnamates and the acetylated compounds oat spelt xylan and xylose tetra-acetate. As shown in Figure 6, LipA exhibited low specificity preference towards short chain *p*NP ester (C_2_) and intermediate activity towards long-chain *p*NP esters (C_14_ and C_16_). Although lipases are capable of hydrolyzing long chain fatty acid esters, some lipases have maximum activity towards medium or shorter acyl groups. LipA had a clear preference for medium chain fatty acid ester (*p*NP caprylate, C8) (Figure 6) demonstrating substrate specificity similar to that obtained with other lipases from microorganisms such as *Bacillus stearothermophilus* L1 [15] and *Aeromonas* sp. LPB 4 [16]. The enzyme showed a very broad activity toward *p*NP esters with the exception of *p*NP arabinopyranoside, *p*NP arabinofuranoside and *p*NP xylopyranoside (Table 1), illustrating its specificity towards the hydrolysis of acyl esters but no activity towards aromatic residues. No activity was found towards methyl cinnamate suggesting that this enzyme has no hydroxycinnamoyl esterase properties (Table 1).

**Table 1:** Substrate specificity of the purified LipA on various substrates

**Substrate**	**Specific activity**^**a **^**(U/mg)**
**Xylose tetra-acetate**	58.84 ± 2.32
**Oat spelt xylan**	14.12 ± 4.34
***p*NP acetate (C**_**2**_**)**	0.14 ± 0.03
***p*NP butyrate (C**_**4**_**)**	2.80 ± 0.04
***p*NP caprylate (C**_**8**_**)**	6.52 ± 0.13
***p*NP myristate (C**_**14**_**)**	2.56 ± 0.17
***p*NP palmitate (C**_**16**_**)**	1.29 ± 0.02
***p*NP arabino-pyranoside**	ND ^b^
***p*NP arabino-furanoside**	ND
***p*NP xylopyranoside**	ND
**Methyl ferulate**	ND
**Methyl cafeate**	ND
**Methyl sinapinate**	ND
**Methyl *p*-coumarate**	ND

^a^ Unit definition: 1 unit of activity is the amount necessary to hydrolyze 1.0 μmol of substrate per minute at 40°C and pH 7.0. Values are the mean ± the standard deviation (SD) of three independent experiments. ^b^ ND = not detectable. Deacetylation of xylose tetra-acetate and oat spelt xylan was evaluated by determining the amount of acetyl group released per milligram of protein and the hydrolysis of *p*NP substrates was expressed relative to the amount of *p*-nitrophenol liberated pre milligram of LipA.

The kinetic analysis of LipA was determined in 100 mM phosphate buffer (pH 8.0) at 40°C over a concentration range of 0.1 to 1 mM of *p*NP substrates. LipA showed lower affinity towards short fatty acid esters (*p*NP acetate, K_m_ 0.15 mM) and as expected, higher affinity was observed for esters with medium and long carbon chain (Table 2).

**Table 2:** **Kinetics parameters and/or specific activities of LipA on *p*NP substrates**

	**Km (mM)**	**V**_**max **_**(μmol/min/mg)**	**Specific Activity at 0.2 mM (μmol/min/mg)**
***p*NP acetate (C**_**2**_**)**	0.15 ± 0.00*	0.24 ± 0.06	0.14 ± 0.03
***p*NP butyrate (C**_**4**_**)**	0.16 ± 0.00	4.73 ± 0.07	2.80 ± 0.04
***p*NP caprylate (C**_**8**_**)**	0.10 ± 0.00	9.45 ± 0.20	6.52 ± 0.13
***p*NP myristate (C**_**14**_**)**	0.07 ± 0.00	3.42 ± 0.24	2.56 ± 0.17
***p*NP palmitate (C**_**16**_**)**	0.10 ± 0.00	1.84 ± 0.04	1.29 ± 0.02

*Values are means ± SD (*n* = 3).

Although the results using *p*NP substrates indicate that LipA has a higher activity towards medium chain fatty acid esters (Figure 6), LipA demonstrated acetylxylan esterase activity against xylose tetra-acetate (58 U/mg, Table 1) that was similar or higher than the acetylxylan esterase of *Bacillus purmilus* (37 U/mg) [17] and *Fibrobacter succinogenes* Axe6A (11.4 U/mg) and Axe6B (1.3 U/mg) [18]. LipA also showed similar or higher specific activity against acetylated xylan (14 U/mg, Table 1) when compared with acetylxylan esterases of bacterial species: *S. flavogriseus* 45-CD (12 U/mg) and *S. olivochromogenes* (17 U/mg) [19], and *Thermotoga maritime* AxeA (0.7 U/mg) [20].

## Discussion

The quest for new microbial lipases and more cost-effective ways to produce them has received increasing attention because of their potential use as industrial biocatalysts in diverse sectors. With the explosion of genomics, the number of sequenced genes encoding enzymes has increased 14 fold in eight years. However, when compared with the number of enzymes that have been biochemically characterized during the same period, this number has increased only around 3% [21]. Through analysing the genome of the recently sequenced alkaliphilic fungus *Acremonium alcalophilum,* we identified a putative lipase, *lipA*. Lipases belong to a group of enzymes defined as carboxylesterases. This group of enzymes is well known for its activity towards substrates with a range of different sizes of carbon chains and although non-specific esterases are more active on short acyl chain esters, lipases have the capability to catalyze the hydrolysis of carboxylic ester bonds of water-insoluble long chains including natural fat [4].

To characterize *lipA*, the heterologous production of LipA was tested in *A. niger* and transient expression in *N. benthamiana*. Attempts to produce LipA in *A. niger* were unsuccessful. While *A. niger* is known to be one of the most potent producers of extracellular enzymes, its ability to produce heterologous proteins is limited. The high level of proteases in the extracellular fluids can negatively impact the production of heterologous proteins [22]. Also, *A. niger* is a producer of organic acids at the industrial scale and prefers to grow in an acidic environment. Almost all extracellular enzymes of *A. niger* are active in acidic pH [23]. It is possible *A. niger* does not provide a suitable environment to produce enzymes that are active in neutral and alkaline pH, owing to differences in protein folding and/or sensitivity to proteases. *Nicotiana benthamiana,* on the other hand*,* accumulated high levels of LipA, possibly because the enzyme was retained in the ER where conditions are conducive for proper folding, and the pH is in the neutral range [24], enabling accumulation, purification and characterization of the enzyme. These characteristics of plants for the production of heterologous enzymes from organisms that thrive in neutral or high pH might prove extremely useful in future production efforts.

The use of plants as bioreactors has gained increasing interest in the past few years as they offer several advantages over other heterologous systems, including low cost biomass production, considerable scale up potential and the ability to undertake complex post-translational modifications that are essential for the stabilization and/or functionality of some proteins [25]. Our results confirm the usefulness of plants as bioreactors for the production of eukaryotic proteins. Moreover, even though the simplicity of culturing and genetic engineering of *Escherichia coli* makes it a preferable choice as an expression system for screening of candidate proteins, the lack of posttranslational modification mechanisms in this host such as glycosylation, protein maturation and limited formation of disulfide bonds, makes it an unsuitable system for large scale glycoprotein production. With that in mind, plant expression systems can play an important role in heterologous expression of proteins that are difficult to express in microbial hosts.

Lipases also have a variety of other biotechnological applications in diverse industrial sectors including the textile, food, pulp and paper, fat and oleochemical, pharmaceutical and in particular, the detergent industry where an estimated 1000 tons of lipases are added to approximately 13 billion tons of detergents each year [5]. The optimal activity at pH 8.0 and broad thermostability (20 to 50°C) of LipA are characteristics highly desirable to the detergent industry, where enzymes are used as additives to detergents at low temperatures and high pH [26].

Functional analysis of a hypothetical, *in silico* annotated, protein often will start with known substrates for biochemical enzymatic characterization. Generally, the biochemical characterization of lipases in the literature is analyzed using *p*NP esters as the substrate [10]. However, lipases are well known for their broad specific activity, and in some cases are active on more than 50 different ester linked substrates [27], including hydroxycinnamoyl and carbohydrate esters. Biochemical characterization of LipA showed a broad specificity towards *p*NP esters of various carbon chain lengths including the long-chain *p*NP palmitate, a typical lipase substrate but also towards acetylated substrates. Since carboxyl and carbohydrate esterases are from the same α/β hydrolase superfamily and most often share the same catalytic domain, the structural analysis and site-directed mutagenesis of LipA should be performed in the future to evaluate the importance of enzyme structure, binding site and catalytic triad on both esterase activities.

The hemicellulose and pectin structures of plant cell walls are decorated with a variety of side chains that are attached to their backbone, including acetyl groups. These acetyl groups specifically can represent up to 4.32% of dry weight in a variety of crop species [28]. Deacetylation of aspen wood and wheat straw, with up to 90% acetyl ester removal, has indicated that as the xylan becomes increasingly deacetylated, it becomes 5–7 times more digestible [29]. Thus, the discovery and characterization of enzymes that can facilitate the decomposition of these polysaccharides could improve the viability of the cellulosic ethanol industry. Since the plant-produced LipA showed activity against both acetylated xylan and xylose tetra-acetate, the results obtained in this study indicate that LipA has the potential to be used as a supplement to enzymatic cocktails for the hydrolysis of lignocellulosic biomass.

Synergism between xylanases and acetyl xylan esterases has been reported to dramatically improve the release of sugars from xylan and glucan [30-32]. Since the acetylxylan esterase activity of LipA was confirmed on oat spelt xylan and xylose tetra-acetate, it is reasonable to suggest that LipA could be used in combination with other xylanases for future development of biological pretreatment for the cellulosic bioproducts industry and should be further investigated. Additionally, considering that the use of plants to produce industrial proteins is projected to have a cost 10-fold lower than microbial based production system, assuming that the foreign protein accumulates to 10% of TSP [33] and that with cost-sensitive applications, such as biofuels, the possibility of cost reduction to produce a key component for plant cell wall deconstruction may be crucial to make biofuels economically viable, these results represent an important step in making this enzyme commercially available for industrial purposes such as the cellulosic ethanol production.

## Conclusions

The results described here show that the *Acremonium alcalophilum* lipase LipA is a glycoprotein that was efficiently expressed in *N. benthamiana*. The enzyme is very versatile exhibiting both acetylxylan esterase and lipase activities. Moreover, the LipA purified and characterized here has properties potentially useful for diverse industry sectors and its applications should be further developed.

## Methods

### Amplification of the *lipA* gene

The protein sequence of the *Acremonium alcalophilum* genome v2.0, http://genome.jgi-psf.org/Acral2/Acral2.home.html, was analyzed for extracellular biomass-degrading enzymes. SignalP v3.0, http://www.cbs.dtu.dk, was used to detect the presence of secretory signal peptide and additional protein domains were examined using the integrated protein signature databases and tools at the European Bioinformatics Institute, http://www.ebi.ac.uk/Tools/pfa/iprscan/. The *A. alcalophilum* protein with the ID 1062717 was found to possess both a secretory signal peptide and a lipase domain, and we called it LipA (for the first identified lipase from this organism) and its encoding gene as *lipA*. Complementary DNA was synthesized [34] using RNA prepared from *A. alcalophilum* strain JCM 7366 cultured in a mixture of alfalfa and barley straw. Gene-specific primers with the Gateway® recombination sequences were used to PCR-amplify the *lipA* gene using synthesized cDNA as template. The forward and reverse primers used were, respectively, 5′-GGGGACAAGTTTGTACAAAAAAGCAGGCTATGTTGGGCTTCGCCTCGCT-3′ and 5′-GGGGACCACTTTGTACAAGAAAGCTGGGTTCAGCCTTCAGCCGATATTG-3′.

### Plant expression vectors

The amplified *lipA* gene was cloned into the Gateway® donor vector pDONR/Zeo™ (Invitrogen, Carlsbad, USA) and the integrity of the construct was validated by sequence analysis. Using the Gateway cloning system, the *lipA* gene was subsequently subcloned into the pCaMGate-ER plant binary expression vector (Conley et al., in preparation), a variation of pCaMterX [35], a binary vector that places the gene of interest under control of the double-enhanced cauliflower mosaic virus 35S promoter [36] and the nopaline synthase (nos) terminator [37]. The pCamGate-ER vector harbors the tCUP translation enhancer [38], the Pr1b secretory signal peptide from *Nicotiana benthamiana*[39] and Xpress™ tag, *att*R1, followed by the *ccd*B gene, *att*R2, C-Myc detection/purification tag, and a KDEL ER-retrieval signal (Figure 2).

### Protein production in *N. benthamiana* leaves and total soluble protein extraction

A suspension of *Agrobacterium tumefaciens* strain EHA105 carrying the expression construct was mixed with an equal amount of *Agrobacterium* culture containing the suppressor of post-transcriptional gene silencing p19 from cymbidium ringspot virus (CymRSV) [12]. The suspension was co-infiltrated into leaves of 5–6 week old *N. benthamiana* plants through the stomata of abaxial leaf epidermis using a syringe [40]. Infiltrated plants were maintained in a controlled growth chamber for 4 days at 22°C, with a 16 h photoperiod. Five leaf disks (7 mm) from infiltrated tissue were collected and ground in liquid nitrogen using 2.3 mm ceramic beads (BioSpec Products, 11079125z, Bartlesville, USA) in a TissueLyser (Qiagen®). Total soluble protein was extracted from the ground tissue in ice-cold phosphate-saline buffer (PBS), pH 7.4 supplemented with 0.1% Tween-20, 2% PVPP (polyvinyl polypyrrolidone), 1 mM EDTA (ethylenediaminetetraacetic acid), 100 mM sodium acorbate, 1 mM PMSF (phenylmethylsulfonyl fluoride) and 1 μg/ml leupeptin. Protein extraction from a control plant infiltrated only with p19 as a negative control was also performed under similar conditions. Total soluble protein (TSP) concentration was determined using the Bradford assay [41] with bovine serum albumin as standard.

### Determination of molecular mass and quantification of LipA protein production levels

The molecular mass of the enzyme was determined by gel electrophoresis. Plant protein extract was separated by sodium dodecylsulphate-polyacrylamide gel electrophoresis (SDS-PAGE) (10%) and transferred to PVDF membrane. To detect the recombinant protein the membrane was incubated with primary mouse anti-C-myc monoclonal antibody (Genscript, A00864, Piscataway, USA). The primary antibody was detected with HRP-conjugated goat anti-mouse IgG antibody (Bio-Rad, 170–6516, Hercules, USA) and visualized using the ECL detection kit (GE healthcare, Mississauga, Canada) and autoradiography as described by the manufacturer.

Western blots were analyzed using image densitometry with TotalLab TL100 software (Nonlinear Dynamics, Durhan, USA). Intensities were determined by comparison with known amounts of a synthetic positive control protein containing a cellulose-binding domain and a C-myc tag (synthesized by Genscript, Piscataway, USA).

Samples were also analyzed by staining SDS-PAGE gels with Gel-Code Blue reagent (PIERCE, 24590). The amount of protein was determined by comparing to known amounts of BSA loaded on the same gel, using image densitometry with TotalLab TL100 software (Nonlinear Dynamics, Durhan, USA).

### Protein purification

Total soluble protein was extracted from plants producing LipA four days post infiltration (dpi) as described above and purified by affinity chromatography using a c-Myc tagged Protein MILD PURIFICATION KIT (MBL, 3305, Woburn, USA) according to the manufacturer’s instructions.

### Deglycosylation analysis

Enzymatic deglycosylation of transiently produced LipA was carried out on purified protein using PNGase F (Sigma-Aldrich, G5166, St. Louis, USA) according to the manufacturer’s instructions. PNGaseF cleaves all high-mannose, hybrid, and complex-type oligosaccharides from *N*-linked glycoproteins, except for those glycans containing a core α(1,3)-linked fucose residue. The digestion was carried out at 37°C for 3 h followed by SDS-PAGE analysis under reducing conditions and western-blot analysis with anti-C-myc antibody. Control samples were treated the same, except that no PNGase F was added.

### Enzyme assays

LipA activity was determined by measuring *p*-nitrophenol (*p*NP) release from *p*-nitrophenyl caprylate [42,43]. The reaction was carried out in 96 well flat bottom microplates using 1 mM *p*-nitrophenol ester substrate in 100 mM phosphate buffer pH 8.0 incubated at 40°C for 30 min. Purified enzyme was added to the reaction and samples were kept in the dark to avoid autohydrolysis of the substrate. *p*-nitrophenol in 100 mM phosphate buffer was used as the standard and the color intensity was measured at 405 nm in a Synergy HT microtitre plate reader (Biotek, Winooski, VT, USA). One unit of lipase activity was defined as the amount of enzyme releasing 1.0 μmol of *p*-nitrophenol per minute under assay conditions. The optimum enzyme pH was measured using sodium-acetate (100 mM, pH 4.0-5.0), sodium-phosphate (100 mM, pH 6.0-9.0) and sodium carbonate (100 mM, pH 10.0-11.0) buffers incubated at 40°C for 30 min. The optimum temperature was determined in the range of 20°C to 90°C in 100 mM sodium-phosphate buffer pH 7.0 incubated for 30 min.

### Substrate specificity and kinetics analysis

Substrate specificity was measured by incubating the purified lipase with *p*NP-derived esters of various lengths (*p*NP acetate, C2; *p*NP butyrate, C4; *p*NP caprylate, C8; *p*NP myristate, C14; *p*NP palmitate, C16) and measuring the amount of *p*NP released. One unit of esterase/lipase activity was defined as the amount of enzyme required to release 1 μmol/min at 40°C. To define the enzyme kinetics, specific activity and kinetic constants were measured using the *p*NP derived ester over eight different substrate concentrations (between 10 μM and 1 mM) to determine the initial reaction rate of the enzyme.

Hydroxycinnamate esters (methyl ferulate, methyl caffeate, methyl *p*-coumarate and methyl sinapinate) were also evaluated as substrates by measuring the release of the corresponding phenolic acids (ferulic acid, caffeic acid, *p*-coumaric acid and sinapinic acid). The 150 μl reaction of 0.2 mM substrate in 100 mM sodium phosphate buffer, pH 7.0 was used to measure the absorbance variation at 335 nm in the Synergy HT plate reader (BioTek®, Winooski, VT, USA). The result of LipA reaction against hydroxycinnamate esters substrate was compared against reactions of feruloyl esterase from *Anaeromyces mucronatus* as positive control under the same conditions.

The liberation of acetic acid from xylose tetra-acetate and oat spelt xylan (Sigma Aldrich, product no. X-0627) was used to quantify acetylxylan esterase activity using the R-Biopharm enzymatic analysis kit (Darmstadt, Germany, Cat no. 10148261035) according to manufacturer’s instructions. The assays were performed in sodium phosphate buffer (50 mM, pH 8.0).

## Abbreviations

LipA: Gene encoding LipA protein from *A. alcalophilum*; Axe: Acetylxylan esterase; TSP: Total soluble protein; SDS-PAGE: Sodium dodecylsulphate-polyacrylamide gel electrophoresis; PNGase: F Peptide -*N*-Glycosidase F; Bp: Base pair; pNP: *p*-nitrophenol; PCR: Polymerase chain reaction; ER: Endoplasmic reticulum; PVPP: Polyvinyl polypyrrolidone; PBS: Phosphate-buffered saline; EDTA: Ethylenediaminetetraacetic acid; PMSF: Phenylmethylsulfonyl fluoride; HRP: Horse radish peroxidase; IgG: Immunoglobulin G; tCUP: Tobacco cryptic upstream promoter; Pr1b: Pathogenesis-related protein 1b of tobacco; ccdB: Gene encoding the cytotoxic protein CcdB.

## Competing interests

The authors declare that they have no competing interests.

## Authors’ contributions

EOP designed the constructs, produced the enzyme, analyzed enzyme activity and drafted the manuscript. TM provided materials, lab space and technical help for activity assays, AT provided the cDNA, and the results in *A. niger*, RM conceived of the study and participated in its design. EOP, TM, AT and RM edited the manuscript. All authors read and approved the final manuscript.

## References

[B1] Okada G Niimura Y Sakata T Uchimura T Ohara N Suzuki H Kozaki M *Acremonium alcalophilum*, a new alkalophilic cellulolytic hyphomycete Trans Mycol Soc Jpn 1993 34 171 185

[B2] Castro-Ochoa LD Rodríguez-Gómez C Valerio-Alfaro G Oliart Ros R Screening, purification and characterization of the thermoalkalophilic lipase produced by *Bacillus thermoleovorans* CCR11 Enzyme Micob Technol 2005 37 648 654 10.1016/j.enzmictec.2005.06.003

[B3] Shangguan JJ Liu YQ Wang FJ Zhao J Fan LQ Li SX Xu JH Expression and characterization of a novel lipase from *Aspergillus fumigatus* with high specific activity Appl Biochem Biotechnol 2011 165 949 962 10.1007/s12010-011-9311-2 21744116

[B4] Chahiniana H Sarda L Distinction between esterases and lipases: comparative biochemical properties of sequence-related carboxylesterases Protein Pept Lett 2009 16 1149 1161 10.2174/092986609789071333 19508178

[B5] Jaeger K-E Reetz MT Microbial lipases form versatile tools for biotechnology Trends Biotechnol 1998 16 396 403 10.1016/S0167-7799(98)01195-0 9744114

[B6] Singh A Mukhopadhyay M Overview of fungal lipase: a review Appl Biochem Biotechnol 2012 166 486 520 10.1007/s12010-011-9444-3 22072143

[B7] Jeoh T Michener W Himmel M Decker S Adney W Implications of cellobiohydrolase glycosylation for use in biomass conversion Biotechnol Biofuels 2008 1 1 12 10.1186/1754-6834-1-1 18471276 PMC2427024

[B8] Ahmad A Pereira EO Conley AJ Richman AS Menassa R Green biofactories: recombinant protein production in plants Recent Pat Biotechnol 2010 4 242 259 10.2174/187220810793611464 21171961

[B9] Zhu S-S Cloning and characterization of two lipases and a lysophospholipase from Aspergillus niger Masters 2007 Biology: Concordia University

[B10] Jaeger KE Dijkstra BW Reetz MT Bacterial biocatalysts: molecular biology, three-dimensional structures, and biotechnological applications of lipases Annu Rev Microbiol 1999 53 315 351 10.1146/annurev.micro.53.1.315 10547694

[B11] Wohlfarth S Hoesche C Strunk C Winkler UK Molecular genetics of the extracellular lipase of *Pseudomonas aeruginosa* PAO1 J Gen Microbiol 1992 138 1325 1335 10.1099/00221287-138-7-1325 1512563

[B12] Silhavy D Molnar A Lucioli A Szittya G Hornyik C Tavazza M Burgyan J A viral protein suppresses RNA silencing and binds silencing-generated, 21- to 25-nucleotide double-stranded RNAs EMBO J 2002 21 3070 3080 10.1093/emboj/cdf312 12065420 PMC125389

[B13] Yuasa K Maeshima M Purification, properties, and molecular cloning of a novel Ca(2+)-binding protein in radish vacuoles Plant Physiol 2000 124 1069 1078 10.1104/pp.124.3.1069 11080284 PMC59206

[B14] Karen AM Kole C, Joshi CP, Shonnard DR In Planta Production of Cell Wall Degrading Enzymes Handbook of Bioenergy Crop Plants 2012 Boca Raton, FL: CRC Press 55 73

[B15] Kim HK Park SY Lee JK Oh TK Gene cloning and characterization of thermostable lipase from *Bacillus stearothermophilus* L1 Biosci Biotechnol Biochem 1998 62 66 71 10.1271/bbb.62.66 9501519

[B16] Lee HK Ahn MJ Kwak SH Song WH Jeong BC Purification and characterization of cold active lipase from psychrotrophic *Aeromonas* sp: LPB 4 J Microbiol 2003 41 22 27

[B17] Degrassi G Okeke BC Bruschi CV Venturi V Purification and characterization of an acetyl xylan esterase from *Bacillus pumilus* Appl Environ Microbiol 1998 64 789 792 10215579 10.1128/aem.64.2.789-792.1998 PMC106121

[B18] Kam DK Jun H-S Ha JK Inglis GD Forsberg CW Characteristics of adjacent family 6 acetylxylan esterases from *Fibrobacter succinogenes* and the interaction with the Xyn10E xylanase in hydrolysis of acetylated xylan Can J Microbiol 2005 51 821 832 10.1139/w05-074 16333341

[B19] Johnson KG Fontana JD MacKenzie CR Measurement of acetylxylan esterase in *Streptomyces* Methods Enzymol 1988 160 551 560

[B20] Tsuchiya A Nakazawa H Toida J Ohnishi K Sekiguchi J Cloning and nucleotide sequence of the mono- and diacylglycerol lipase gene (mdlB) of *Aspergillus oryzae* FEMS Microbiol Lett 1996 143 63 67 10.1111/j.1574-6968.1996.tb08462.x 8807803

[B21] Cantarel BL Coutinho PM Rancurel C Bernard T Lombard V Henrissat B The carbohydrate-active enzymes database (CAZy): an expert resource for Glycogenomics Nucleic Acids Res 2009 37 233 238 10.1093/nar/gkn663 PMC2686590 18838391

[B22] van den Hombergh JPTW van de Vondervoort PJI Fraissinet-Tachet L Visser J Aspergillus as a host for heterologous protein production: the problem of proteases Trends Biotechnol 1997 15 256 263 10.1016/S0167-7799(97)01020-2 9237405

[B23] Murphy C Powlowski J Wu M Butler G Tsang A Curation of characterized glycoside hydrolases of fungal origin Database (Oxford) 2011 2011 bar020 10.1093/database/bar020 21622642 PMC3263737

[B24] Vitale A Denecke J The endoplasmic reticulum-gateway of the secretory pathway Plant Cell 1999 11 615 628 10213782 10.1105/tpc.11.4.615 PMC144197

[B25] Menassa R Ahmad A Joensuu JJ Wang A, Ma S Transient expression using agroinfiltration and its applications in molecular farming Molecular farming in plants: recent advances and future prospects 2012 Dordrecht, Heidelberg, London, New York: Springer Netherlands 183 198

[B26] Margesin R Schinner F Properties of cold-adapted microorganisms and their potential role in biotechnology J Biotechnol 1994 33 1 14 10.1016/0168-1656(94)90093-0

[B27] Martinez-Martinez M Alcaide M Tchigvintsev A Reva O Polaina J Bargiela R Guazzaroni ME Chicote A Canet A Valero F Biochemical diversity of carboxyl esterases and lipases from lake arreo (Spain): a metagenomic approach Appl Environ Microbiol 2013 79 3553 3562 10.1128/AEM.00240-13 23542620 PMC3675924

[B28] Pronyk C Mazza G Fractionation of triticale, wheat, barley, oats, canola, and mustard straws for the production of carbohydrates and lignins Bioresour Technol 2012 106 117 124 22197077 10.1016/j.biortech.2011.11.071

[B29] Grohmann K Mitchell DJ Himmel ME Dale BE Schroeder HA The role of ester groups in resistance of plant cell wall polysaccharides to enzymatic hydrolysis Appl Biochem Biotechnol 1989 20–21 45 6123918082

[B30] Zhang J Siika-Aho M Tenkanen M Viikari L The role of acetyl xylan esterase in the solubilization of xylan and enzymatic hydrolysis of wheat straw and giant reed Biotechnol Biofuels 2011 4 1 10 10.1186/1754-6834-4-1 22185437 PMC3259036

[B31] Huang YC Chen GH Chen YF Chen WL Yang CH Heterologous expression of thermostable acetylxylan esterase gene from *Thermobifida fusca* and its synergistic action with xylanase for the production of xylooligosaccharides Biochem Biophys Res Commun 2010 400 718 723 10.1016/j.bbrc.2010.08.136 20816933

[B32] Selig MJ Knoshaug EP Adney WS Himmel ME Decker SR Synergistic enhancement of cellobiohydrolase performance on pretreated corn stover by addition of xylanase and esterase activities Bioresour Technol 2008 99 4997 5005 10.1016/j.biortech.2007.09.064 18006303

[B33] Hood E Woodard S Hood E, Howard J Industrial proteins produced from transgenic plants Plants as factories for protein production 2002 Dordrecht: Springer Netherlands 119 135

[B34] Semova N Storms R John T Gaudet P Ulycznyj P Min XJ Sun J Butler G Tsang A Generation, annotation, and analysis of an extensive *Aspergillus niger* EST collection BMC Microbiol 2006 6 1 10 10.1186/1471-2180-6-1 16457709 PMC1434744

[B35] Harris LJ Gleddie SC A modified *Rpl3* gene from rice confers tolerance of the *Fusarium graminearum* mycotoxin deoxynivalenol to transgenic tobacco Physiol Mol Plant Pathol 2001 58 173 181 10.1006/pmpp.2001.0326

[B36] Kay R Chan A Daly M McPherson J Duplication of CaMV 35S promoter sequences creates a strong enhancer for plant genes Science 1987 236 1299 1302 10.1126/science.236.4806.1299 17770331

[B37] Bevan M Barnes WM Chilton MD Structure and transcription of the nopaline synthase gene region of T-DNA Nucleic Acids Res 1983 11 369 385 10.1093/nar/11.2.369 6298724 PMC325720

[B38] Wu K Malik K Tian L Hu M Martin T Foster E Brown D Miki B Enhancers and core promoter elements are essential for the activity of a cryptic gene activation sequence from tobacco, tCUP Mol Genet Genomics 2001 265 763 770 10.1007/s004380100478 11523793

[B39] Cutt JR Dixon DC Carr JP Klessig DF Isolation and nucleotide sequence of cDNA clones for the pathogenesis-related proteins PR1a, PR1b and PR1c of *Nicotiana tabacum* cv: Xanthi nc induced by TMV infection Nucleic Acids Res 1988 16 9861 10.1093/nar/16.20.9861 3186451 PMC338789

[B40] Conley AJ Joensuu JJ Jevnikar AM Menassa R Brandle JE Optimization of elastin-like polypeptide fusions for expression and purification of recombinant proteins in plants Biotechnol Bioeng 2009 103 562 573 10.1002/bit.22278 19266472

[B41] Bradford MM A rapid and sensitive method for the quantitation of microgram quantities of protein utilizing the principle of protein-dye binding Anal Biochem 1976 72 248 254 10.1016/0003-2697(76)90527-3 942051

[B42] Qi M Wang P Selinger LB Yanke LJ Forster RJ McAllister TA Isolation and characterization of a ferulic acid esterase (Fae1A) from the rumen fungus *Anaeromyces mucronatus* J Appl Microbiol 2011 110 1341 1350 10.1111/j.1365-2672.2011.04990.x 21362116

[B43] Ghatora SK Chadha BS Saini HS Bhat MK Faulds CB Diversity of plant cell wall esterases in thermophilic and thermotolerant fungi J Biotechnol 2006 125 434 445 10.1016/j.jbiotec.2006.04.005 16713648

